# Effects of low-temperature capping on the optical properties of GaAs/AlGaAs quantum wells

**DOI:** 10.1186/1556-276X-6-76

**Published:** 2011-01-12

**Authors:** Masafumi Jo, Guotao Duan, Takaaki Mano, Kazuaki Sakoda

**Affiliations:** 1 National Institute for Materials Science, 1-2-1 Sengen, Tsukuba, Ibaraki 305-0047, Japan

## Abstract

We study the effects of low-temperature capping (200-450°C) on the optical properties of GaAs/AlGaAs quantum wells. Photoluminescence measurements clearly show the formation of abundant nonradiative recombination centers in an AlGaAs capping layer grown at 200°C, while there is a slight degradation of the optical quality in AlGaAs capping layers grown at temperatures above 350°C compared to that of a high-temperature capping layer. In addition, the optical quality can be restored by post-growth annealing without any structural change, except for the 200°C-capped sample.

## Introduction

Self-assembled semiconductor nanostructures have attracted tremendous interest due to their excellent electronic and optical properties. Since the properties of nanostructures strongly depend on their size, shape, and composition, it is important to reduce the morphological change of nanostructures during the capping process. In this context, much research has recently focused on low-temperature capping with less atomic intermixing, although it is commonly believed that the crystalline quality of the capping layer deteriorates quickly with decreasing temperature.

Droplet epitaxy is a self-assembled growth technique based on the formation of metallic droplets followed by crystallization into semiconductor quantum dots (QDs) [1-13]. Droplet epitaxy allows the self-assembly of QDs in lattice-matched systems such as GaAs/AlGaAs, which is unattainable in a conventional Stranski-Krastanow growth mode. In the growth of GaAs/AlGaAs QDs, various quantum structures such as monomodal dots [3], single/multiple rings [4,8,9], and nanoholes [10-13] have been derived by controlling the As pressure and temperature during the crystallization of Ga droplets.

However, in droplet epitaxy, low-temperature processes at around 200°C are required for the formation of droplets and their crystallization, which often causes degradation of the crystalline and optical qualities of the QDs and subsequent AlGaAs capping layer. Uncapped annealing of QDs is, therefore, used as an effective way to improve the quality of the QDs [14]. This annealing step, however, can also cause significant morphological changes in the QD. For example, GaAs QDs grown on GaAs(001) substrates elongate in the [-110] direction when annealed at temperatures higher than 400°C [15], and so a capping temperature below 400°C is necessary for embedding QDs with their original morphology maintained. However, such a low temperature is challenging for the growth of high-grade AlGaAs, and indeed, the effects of a low-temperature AlGaAs capping layer on the optical properties of adjacent GaAs quantum structures have not yet been clarified.

We studied the optical qualities of GaAs nanostructures capped with a low-temperature AlGaAs layer. To clarify the effects of the capping layer, we used high-quality GaAs/AlGaAs single quantum wells (QWs) capped at various temperatures. Luminescence study showed a clear difference between the sample capped at 200°C and the samples capped above 350°C, which is explained by the incorporation of excess arsenic in the AlGaAs grown at low temperatures (< 300°C).

## Experimental procedures

**Figure 1:**
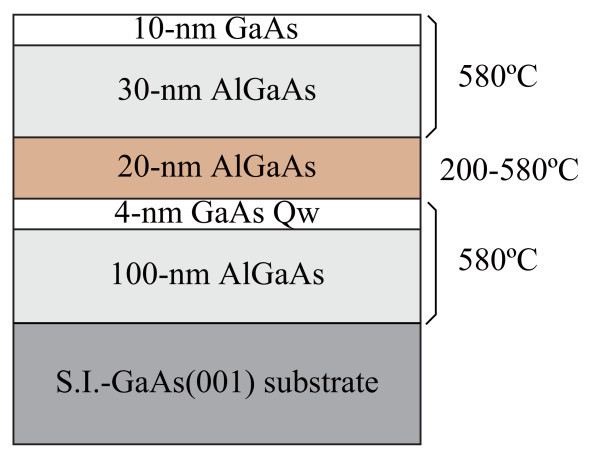
**Sample structure of a 4-nm GaAs/AlGaAs QW**. An AlGaAs capping layer of 20 nm was grown at different temperatures of 200, 350, 450, and 580°C.

Figure 1 shows the sample structure used in this study. High-quality 4-nm GaAs/AlGaAs single QWs were grown on semi-insulating GaAs(001) substrates by molecular beam epitaxy at 580°C. Then the substrate temperature was lowered and the QWs were capped with 20-nm AlGaAs at 200, 350, 450, and 580°C. For the capping at 350, 450, and 580°C, the growth rate was set at one monolayer (ML) per second and As_4 _flux of 2 × 10^-5 ^Torr was used. Only for the capping at 200°C, we used migration enhanced epitaxy (MEE) to assure smooth growth [16]. The MEE sequence consisted of alternative deposition of III-materials and V-materials: Al and Ga for 1 s (1 ML s^-1^) and As for 5 s (2 × 10^-6 ^Torr). Note that the above growth parameters were not optimized. After the first capping, the substrate was heated to 580°C, and second capping layers (30-nm AlGaAs + 10-nm GaAs) were grown at 580°C for all samples. During the growth, the surface state was monitored by reflection high-energy electron diffraction (RHEED). The optical properties of the samples were investigated in terms of photoluminescence (PL). PL spectra were taken at 6 K, using the 532-nm line of a frequency-doubled Nd:YAG laser. The PL signals were dispersed by a monochromator and detected by a cooled Si charge-coupled device array.

## Results and discussion

**Figure 2:**
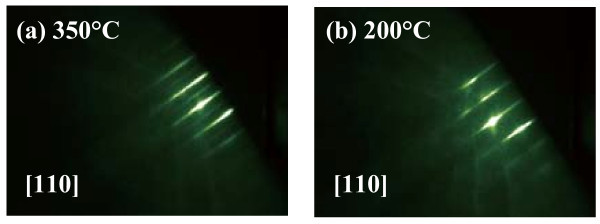
**RHEED patterns of an AlGaAs capping layer grown**. **(a) **at 350°C, and (b) at 200°C.

First, the surface morphology of the AlGaAs capping layer was investigated by RHEED imaging. Figure 2a shows the RHEED pattern of the sample capped at 350°C. The surface exhibits a clear *c*(4 × 4) reconstruction with streaky features, indicating that a flat surface was obtained. When we decreased the capping temperature to 200°C, the diffraction image changed from *c*(4 × 4) to (1 × 1) as shown in Figure 2b. However, the pattern remained streaky, which suggests two-dimensional growth of the capping layer at 200°C.

**Figure 3:**
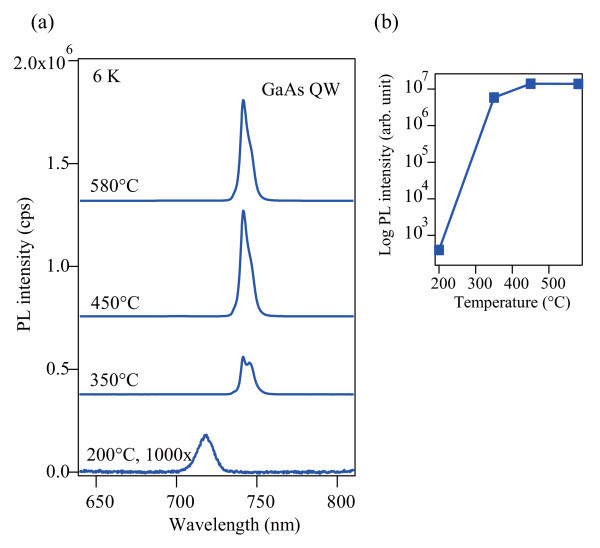
**PL properties of GaAs/AlGaAs QWs**. **(a) **Low-temperature PL spectra of 4-nm GaAs/AlGaAs QWs capped at different temperatures. **(b) **Integrated PL intensity plotted as a function of the capping temperature.

Although a good surface morphology was observed for all samples, the optical quality varied greatly between the samples as shown in Figure 3. Let us first focus on the samples capped above 350°C in which sharp emission lines from the GaAs QWs were obtained. The QW emission around 740 nm consists of two peaks corresponding to different well thicknesses of 14 and 15 MLs, as is clearly resolved in the sample capped at 350°C. A constant linewidth of about 15 meV is observed for all three samples, indicating that both the incorporation of impurities at the interface and local charging effects due to defects in the AlGaAs capping layer are negligibly small. The optical quality of the AlGaAs capping layer can be monitored by the PL intensity of the QW. In the sample capped at 450°C, the PL intensity is almost the same as that of the 580°C sample. Even in the sample capped at 350°C, the intensity still remains at almost 50% of that of the 580°C sample. These results illustrate that reasonably high-quality capping can be achieved above 350°C for the optical emission from QWs, although the number of nonradiative recombination centers might increase slightly at 350°C.

In contrast, the sample capped at 200°C exhibits faint emission around 718 nm, which is blue shifted by 60 meV compared to the QW emission from the sample capped above 350°C. The emission linewidth also increases to 30 meV. We attribute this change to the incorporation of excess As atoms into the AlGaAs capping layer during the low-temperature growth. It is well known that GaAs grown at temperatures below 300°C becomes nonstoichiometric with an excess of arsenic incorporated as a point defect in the GaAs matrix [17,18]. The excess arsenic forms precipitates when annealed at temperatures above 500°C, but the epilayer is still highly nonradiative due to the presence of residual point defects [19] or resultant metallic As clusters [20]. In our case, the AlGaAs capping layer containing As clusters was developed during the subsequent growth of the second capping layer at 580°C. Not only does the annealed low-temperature AlGaAs layer act as a nonradiative pathway, but the As clusters may modulate the QW potential, resulting in the imperceptible emission with a peak shift.

**Figure 4:**
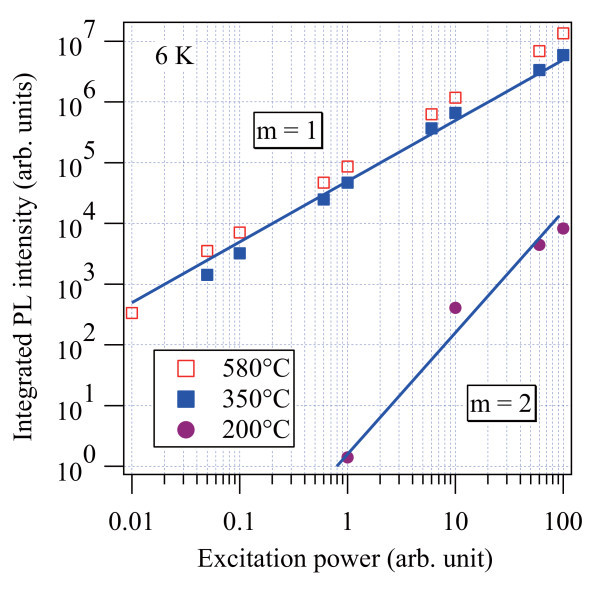
**Excitation power dependence of the integrated PL intensity of the samples capped at 580, 350, and 200°C**. Solid lines denote the linear dependence (*m *= 1) and the quadratic dependence (*m *= 2), respectively.

The differences in optical quality were further studied by the excitation power dependence of the PL. Figure 4 plots integrated PL intensity as a function of the excitation power. The PL intensities of the samples capped at 580 and 350°C increase linearly (*m *= 1) with respect to the excitation power, illustrating that radiative recombination dominates in both samples [21]. On the other hand, the quadratic (*m *= 2) development observed in the 200°C-capped sample is consistent with the fact that the nonradiative decay channels are strongly active in the capping layer.

Here we would like to compare our results with previous reports on the properties of GaAs grown at low temperatures. Since the first report by Stall et al. [22] that the electrical properties of GaAs were degraded when grown below 480°C, many efforts have been made to obtain good quality of GaAs at low temperatures. Metze et al. [23] were able to grow good-quality GaAs at 450°C by reducing the growth rate to 0.2 μm h^-1^. Missous and Singer [24] pointed out the superiority of As_2 _in reducing the concentration of deep levels compared to As_4_. By contrast, our growth condition was "normal", i.e., the growth rate was 1 μm h^-1 ^and an As_4 _source was used. The difference is that the epilayer was very thin and undoped in our case. In fact, our purpose is to embed nanostructures with little atomic diffusion, and the thickness (volume) of the capping layer is very small compared to that of the whole structure. Our results show that a thin capping layer does not significantly lower the quantum efficiency of the embedded nanostructure, even though the capping layer was grown at a low temperature with a normal condition. Of course the quality of the capping layer would be improved by optimizing the growth conditions such as growth rate, V/III ratio, and As species.

**Figure 5:**
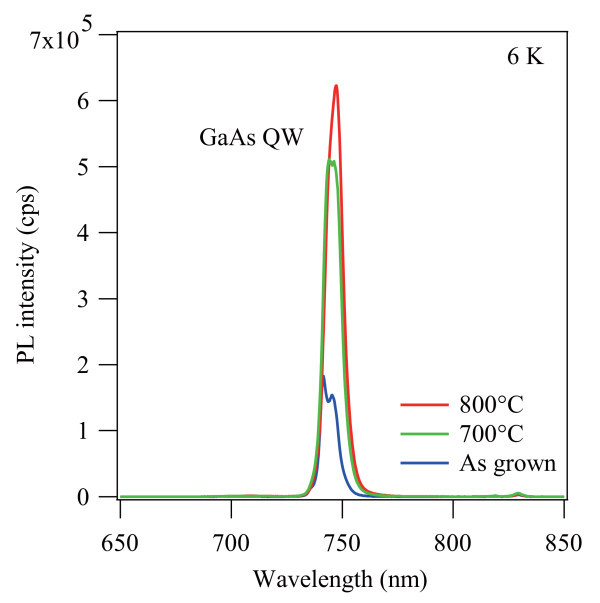
**6-K PL spectra of the 350°C-capped sample annealed at different temperatures**.

Finally, the effect of post-growth annealing was studied. To improve the quality, we performed rapid thermal annealing (4 min, N_2 _ambient) on the 350°C-capped sample. Figure 5 shows PL spectra of the sample annealed at 700 and 800°C, along with the as-grown one. The PL intensity increases with increasing annealing temperature, and eventually becomes equivalent to that of the 580°C-capped sample. Furthermore, the peak position and linewidth remain unchanged during the annealing, indicating no significant intermixing between the GaAs QW and the AlGaAs capping layer. Note that such restoration of sharp emission from the GaAs QW was not observed in the 200°C-capped sample since the excess As atoms are difficult to remove even after post-growth annealing.

## Conclusion

We have studied the effects of a low-temperature AlGaAs capping layer on the optical properties of a GaAs QW, using different capping temperatures of 200, 350, 450, and 580°C. Although a good morphology was obtained for all samples, there was a clear difference in the optical qualities between the 200°C-capped sample and the others. In the sample capped at 200°C, incorporation of excess arsenic followed by the formation of As clusters introduces many nonradiative recombination centers in the AlGaAs capping layer, which greatly reduces the PL from the QW. By contrast, the sample capped above 350°C showed clear emission from the QW, though a slight degradation in intensity was observed with decreasing capping temperature. Except for the 200°C-capped sample, the quality could be restored to that of the 580°C-capped sample without any structural change caused by post-growth annealing at 800°C. These results clearly demonstrate that the capping temperature of 350°C is high enough to obtain a quantum structure with high quantum efficiency, thus paving the way for low-temperature capping of QDs to suppress morphological changes and interdiffusion.

## Abbreviations

ML: monolayer; PL: photoluminescence; RHEED: reflection high-energy electron diffraction; QDs: quantum dots; QWs: quantum wells.

## Competing interests

The authors declare that they have no competing interests.

## Authors' contributions

MJ carried out the optical measurements, participated in the sequence alignment and drafted the manuscript. GD performed the sample growth. TM participated in the design and coordination of the study. KS participated in the design of the study. All authors read and approved the final manuscript.
